# Academy of Stomatology

**Published:** 1894-11

**Authors:** 


### ACADEMY OF STOMATOLOGY.

THE Academy met at the office of Dr. Darby, 1513 Walnut Street, Philadelphia, October 6, 1894.

The meeting was called to order by the President, Dr. Louis Jack.

The monthly routine business of the Society having been arranged, the essayist of the evening, Dr. M. H. Cryer, was requested by the President to present his paper, together with the proposed demonstrations.

(For Dr. Cryer's paper, see page 699.)

At the conclusion of the reading a patient was presented, and the operation previously performed explained as follows:

"The patient reported at my dispensary in April with what appeared to be a large alveolo-dental abscess. Upon examination with an exploring needle I found I could penetrate to its full length without striking the bone. She was dismissed for a few days to allow time for consideration. Upon the second visit I made a microscopic examination of a small section of the tumor, and it proved to be a giant-cell sarcoma. Reporting the case to Professor Garretson, surgeon in charge, it was decided that there was but one course to pursue, and that was to remove it as speedily and as thoroughly as possible."

The position of the tumor was then indicated upon the patient present, together with the mode of operating with the various instruments, including the use of the surgical engine. The description was then resumed:

"After consultation with Professor Garretson, who is surgeon in charge, the tumor was removed and the bone made thoroughly smooth with the engine-burs; the finger was passed over it to insure that nothing but healthy bone remained. It is not difficult to distinguish healthy from unhealthy bone by the touch of the instrument.

"When the diseased bone was removed, the parts carefully syringed, and the severe hemorrhage stopped, the parts were packed with iodoform gauze as tightly as possible. The lip was then turned down to its proper position, and three hare-lip pins were placed to hold it in position below the nose, and five or six sutures around the alæ. On the Monday following the upper pin was removed, and on Tuesday the lower pin; on Wednesday the remaining one and a few of the stitches were taken out, and at the same time some of the iodoform gauze. By the following Saturday the balance of the iodoform gauze was removed and the patient was practically well, but was detained a week longer. There is some mutilation, of course, in the mouth, being obliged to sacrifice several teeth, but in other respects no special disfigurement can be observed."

The case was then presented for the examination of the members present.

Dr. Cryer then gave demonstrations on the heads of sheep and human skulls of his method of operating with the new improved surgical engine, proving very satisfactorily to those present that by its use, with the improved burs, it was far in advance of the old methods, admitting of more thorough work, with greater speed and with less pain and mutilation of parts.

At the conclusion of some further general business the President called for

INCIDENTS OF PRACTICE.

Dr. Gaskill referred to a case previously reported and remarked: "For the benefit of those not acquainted with this case, I may repeat that the patient presented about two and a half years ago with a very marked condition of absorption of the tissues of the tooth, evidently proceeding from the pulp without external opening. In time the enamel and dentine were crushed down on the palatine surface. About two years subsequent to this the adjoining central incisor presented the same appearance, exhibiting the pulp through the wall of enamel. I placed the patient under the treatment of iodide of arsenic and Fellows' hypophosphites, at the suggestion of Dr. Kirk, with excellent results, as there is now a deposition of dentine in the enlarged pulp-chamber, mainly obliterating the spot on the labial wall. It is still evident, but greatly reduced. The patient reports periodically for examination.

Dr. Truman stated that he had recently received a paper of much interest from Dr. White, of New Mexico, on the use of antiseptics, and accompanying this was sent a sample of balsam, called by the natives "balsamo del deserto." The full description of this new agent will be found in the present number of the International Dental Journal.

The success which had attended the use of this for several years in the filling of root-canals had led Dr. White to regard it of great value, and he had therefore forwarded several samples with the request that it might be tested more generally, in the hope that his conclusions as to its value might be confirmed. The time had been too short since these samples had been received to give a practical test, but should Dr. White's conclusions be confirmed, and theoretically there is no reason why they should not be, we may find in this balsam a most valuable addition to the few materials now possessed for this purpose of canal filling, and possibly superior to all of them.

Extracts were read from Dr. White's letters, the main points of which are to be found in his paper.

Dr. Kirk said, "There is one thought that occurs to me. From correspondence and conversation with dentists in tropical and semitropical countries, I am led to believe that the difficulties met with there are quite different from those we are obliged to overcome here. In other words, pericemental inflammations are much more likely to occur under our climatic conditions, unless the treatment be more thorough than Dr. White has described. With his mode of managing such cases we would certainly have trouble."

Dr. Truman stated that he had no intention of advocating the use of the balsam, but it seemed to him to have a special value, and as it was antiseptic and impervious to moisture and practically indestructible, it appeared as though it might yet fill an important place in our offices. If as represented, it will prove a great boon in the treatment of children's teeth.

Dr. Jack suggested that perhaps much of its value might be ascribed to the oil of cinnamon combined with it, as described by Dr. White.

Dr. Kirk said that there was a great need of a suitable filling for the canals in children's teeth. He thought this substance might prove valuable for this purpose. He mentioned a preparation of Dr. Van Woert, of Brooklyn, N. Y., composed of iodol and oxide of zinc, for a similar purpose.

Dr. Darby introduced some fine pulp-instruments made from piano wire. The temper was such that they could be introduced into the most tortuous canals without danger of breaking. With these he could readily remove the pulp from any canal.

Adjourned.

